# Sequence Analysis of *Plum pox virus* Strain C Isolates from Russia Revealed Prevalence of the D96E Mutation in the Universal Epitope and Interstrain Recombination Events

**DOI:** 10.3390/v10090450

**Published:** 2018-08-23

**Authors:** Anna Sheveleva, Peter Ivanov, Tatiana Gasanova, Gennady Osipov, Sergei Chirkov

**Affiliations:** 1 Department of Virology, Faculty of Biology, Lomonosov Moscow State University, Moscow 119234, Russia; anncsh@yandex.ru (A.S.); pivanov@genebee.msu.ru (P.I.); tv.gasanova@gmail.com (T.G.); 2 Tatar Research Institute of Agriculture, Kazan 420059, Russia; osipovge@mail.ru

**Keywords:** *Plum pox virus*, sour cherry, strain C, next-generation sequencing, recombination, seed transmission

## Abstract

The understanding of genetic diversity, geographic distribution, and antigenic properties of *Plum pox virus* (PPV) is a prerequisite to improve control of sharka, the most detrimental viral disease of stone fruit crops worldwide. Forty new PPV strain C isolates were detected in sour cherry (*Prunus cerasus*) from three geographically distant (700–1100 km) regions of European Russia. Analysis of their 3’-terminal genomic sequences showed that nineteen isolates (47.5%) bear the D96E mutation in the universal epitope of the coat protein. Almost all of them cannot be detected by the monoclonal antibody 5B in triple antibody sandwich enzyme-linked immunosorbent assay and Western blot analysis that may potentially compromise serological PPV detection in cherries. Full-length genomes of seven PPV-C isolates were determined employing next-generation sequencing. Using the Recombination Detection Program (RDP4), the recombination event covering the region from (Cter)*P1* to the middle of the *HcPro* gene was predicted in all the available PPV-C complete genomes. The isolates Tat-4, belonging to the strain CV, and RU-17sc (PPV-CR) were inferred as major and minor parents, respectively, suggesting possible pathways of evolution of the cherry-adapted strains. Downy cherry (*P. tomentosa*) was identified as the natural PPV-C host for the first time.

## 1. Introduction

*Plum pox virus* (PPV, genus *Potyvirus*, family *Potyviridae*) is the causal agent of sharka, the most detrimental viral disease of stone fruit crops [1]. It has a single stranded positive sense RNA genome about 10 kb in length encoding a 355 kDa polyprotein. A short overlapping open reading frame (ORF), called PIPO, is also expressed as a P3N-PIPO fusion product essential for cell-to-cell movement [2]. PPV is transmitted from plant to plant through vegetative propagation of infected material and by aphids in a non-persistent manner. The virus was found worldwide except Australia, New Zealand, and South Africa [3]. One of the reasons for the global spread is apparently the high genetic variability of PPV ensuring its adaptation to diverse stone fruit species and environments. Based on differences in complete genome sequences and phylogenetic analysis, ten PPV strains (D, M, C, EA, Rec, W, T, An, CR, CV) are recognized to date [2,4,5]. They may differ in antigenic and epidemiological properties, geographical distribution, pathogenicity, and host preference, although clear links between such features and molecular affiliation is not evident for each strain [6]. Under natural conditions, PPV-C, -CR, and -CV were only found on sour (*Prunus cerasus*) and sweet (*P. avium*) cherries and considered the cherry-adapted strains.

The strains CR and CV have so far been detected only in Russia [5,7,8]. PPV-C was identified in the countries of the former USSR: Moldova [9,10,11], Belarus [12], and Russia [13,14,15,16,17]. In addition, it was reported on sour and sweet cherries from Italy [18], Romania [19], Hungary [20], Croatia [21], and Germany [22]. In most cases, these isolates were found in single trees and the epidemiological relevance of PPV-C was not shown until now. Surveys performed in regions of high PPV-C incidence can improve understanding of the epidemiology of this strain.

Unfortunately, most detected PPV-C isolates have not been characterized to date. Only four complete genome sequences of natural isolates from Belarus (BY101, BY181), Russia (Volk143), and Germany (GC27) have been deposited in GenBank. The full-length genomes of two more isolates SoC and SwC were sequenced after their maintenance in *Nicotiana benthamiana* [23] and may contain mutations resulting from adaptation to the herbaceous host. The insufficient number of available PPV-C sequences limits the capability to analyze their diversity, evolution, and genetic factors contributing to the adaptation to cherry [22].

Sweet and sour cherries are economically important in many regions of the world and especially popular in Europe, the Middle East, the Mediterranean countries, and North America [24]. The PPV-C infection can affect the yield and quality of the fruits. Premature fruit drops and apical necrosis of branches were also observed on naturally infected sweet cherry trees [25]. Experimentally, PPV-C was mechanically, graft, or aphid transmitted to plum (*P. domestica*), peach (*P. persica*), mahaleb cherry (*P. mahaleb*), myrobalan (*P. cerasifera*), apricot (*P. armeniaca*), downy cherry (*P. tomentosa*), *P. marianna*, and some interspecies hybrids [9,18,25,26], posing a potential threat of the PPV-C spreading from infected cherry to other *Prunus* species in the vicinity [27]. In this regard, the study of the prevalence and natural host range of PPV-C to minimize its further dissemination is of great interest.

Enzyme-linked immunosorbent assay (ELISA) test using the PPV-specific monoclonal antibody 5B [28] is considered the most reliable method of the serological detection of the virus [2]. The antibody 5B recognizes the universal epitope ^94/96^DRDVDAG^100/102^ that is localized at the junction of the N-terminal and core domains of the coat protein (CP) and was thought to be expressed in any PPV isolate [29]. However, almost all known isolates of the recently discovered strain CR have been shown to bear the D96E mutation in the universal epitope. These were not detected by triple antibody sandwich (TAS)-ELISA using the antibody 5B [7] or can still be recognized by this antibody after lowering the pH of the extract [8]. Two PPV-C isolates were also reported to bear this mutation and could not be detected using the antibody 5B [14]. Study on the frequency of this mutation among PPV-C isolates is an important challenge to ensure reliable diagnosis of the cherry-adapted isolates.

In this work, we detected and characterized forty new PPV-C isolates collected from cherries in three geographically distant regions of European Russia expanding information on the spread and natural host range of this strain. Analysis of partial and complete genome sequences allowed to detect a broad distribution of the D96E mutation among new isolates and to reveal a new recombination event in PPV-C genomes, thus raising the question of reliable serological PPV detection in cherries and possible pathways of evolution of the cherry-adapted strains.

## 2. Materials and Methods

### 2.1. Cherry Plants and Virus Isolates

Sour cherry leaves displaying typical symptoms of sharka disease (irregular pale green rings, spots, or arabesques) were sampled from adult trees in working and abandoned cultivar collections and cultivar or hybrid test plots in Pavlovsk Research Station of Vavilov Research Institute of Plant Industry (Pavlovsk town, St. Petersburg region, Russia), Botanical Garden of Lomonosov Moscow State University (Moscow, Russia), and Zonal Research Station of Tatar Research Institute of Agriculture (Kazan town, Republic of Tatarstan, Russia) during surveys conducted in 2012–2016. The plantings surveyed are situated in three geographically distant (700–1100 km) regions of European Russia (Figure S1). The isolates studied in this work are listed in Table 1. The Moscow isolates Bg6, Bg10, Bg26, Bg60, and Bg66 were detected on grafted sour cherry plants. All other isolates were collected from own-rooted (nongrafted) trees. Isolates Ka5, Ka10, Ka11, Ka21, Ka55, Ka56 were taken from seed-borne hybrids obtained through free pollination of local cherry cultivars. The isolates Ka19 and Ka20, as well as Ka42–Ka45, were found on rooted cuttings obtained from two different mother plants. The Tatar isolate Ka7 was detected on the seed-borne hybrid of two local cultivars. The isolates Ka1 and Pul were collected from wild sour cherry trees and Ka31, Ka54, Ka57, Ka58 were revealed on root offshoots of unknown origin. Another isolate Ka15 was collected from downy cherry (*P. tomentosa*) displaying mild chlorosis on mature leaves. All virus isolates were kept as lyophilized material of infected leaves in tightly sealed vials at 4 °C.

### 2.2. Enzyme-Linked Immunosorbent Assay (ELISA)

The samples were analyzed by double antibody sandwich (DAS) ELISA using a reagent set SRA 31505 (Agdia, Elkhart, IN, USA) and TAS-ELISA with the monoclonal antibody 5B supplied with a K-10B kit (Agritest S.r.l, Valenzano, Italy). The leaf tissue was grinded in phosphate-buffered saline (PBS) supplemented with 0.02% (*v*/*v*) Tween 20, 2% (*w*/*v*) polyvinylpyrrolidone (MW of about 40,000), 0.5% (*v*/*v*) Triton X-100 and 0.02% (*w*/*v*) sodium azide using 1/20 ratio (*w*/*v*). The clarified extracts were placed in the microplate wells (MaxiSorp, Nunc, New York, NY, USA) pre-coated with the polyclonal PPV-specific antibodies (Agdia) and incubated for 2 h at 37 °C. The subsequent steps were performed according to the kit/set manufacturer’s instructions. Positive and negative controls were taken from the kit K-10B. The optical densities were measured 40 min after substrate addition at the wavelength of 405 nm [30].

### 2.3. Reverse Transcription-Polymerase Chain Reaction (RT-PCR)

Immunocapture RT-PCR was performed according to Wetzel et al. [31] using the plant extracts prepared in the same way as for ELISA (see above). Polyclonal PPV-specific antibodies from a reagent set SRA 31505 (Agdia) were diluted 1/200 in 0.05 M carbonate buffer, pH 9.6, and incubated in disposable micro centrifuge tubes (Multiply-Pro cup 0.5 mL, Sarstedt, Nümbrecht, Germany at 4 °C overnight. Leaf extracts (100 μL) were incubated with the pre-coated antibodies 1 h at 37 °C. The tubes were washed three times with PBS containing 0.05% (*v*/*v*) Tween 20 before and after incubation of plant sap. Genomic RNA was released from immunocaptured virus particles by treatment of tubes with 1% (*v*/*v*) Triton X-100 at 70 °C for 10 min. Random hexamer primers and MMLV reverse transcriptase (Evrogen, Moscow, Russia) were used for the first strand cDNA synthesis. RT products were employed in PCR for (i) PPV detection using the generic primers P1/P2 [32]; (ii) strain identification; and (iii) sequencing of the 3’-terminal genomic region. Strain typing was done using primer pairs HSoC-1/CSoc-1, HSoC-2/CSoC-2 [25,33], and M10-5’/M11-3’ [34] developed for the strain C identification as well as primers to the strains D, M, W, CR, and CV using reaction conditions described in the original protocols [5,8,35,36]. The isolates 1410 [37], Fl-3 [7], Bg66 [14], Pav-17 [38], and Tat-2 [30] from our laboratory collection were used as positive controls for the strains W, CR, C, D, and CV, respectively. The PPV-M positive control was provided with K-11B kit (Agritest). The negative control was obtained from PPV-free sour cherry leaves. PCR products were analyzed by 2% (*w*/*v*) agarose gel electrophoresis and visualized by ethidium bromide staining.

### 2.4. Sequencing of the 3’-Terminal Genomic Region

The 3’-terminal genomic region spanning the entire *CP* gene and flanking sequences of the *NIb* gene and 3’-non-coding region was amplified employing the forward primer p84 [23] and the reverse primer 4CPR1 [39]. PCR included denaturation at 94 °C for 30 s, primer annealing at 60 °C for 30 s, and extension at 72 °C for 1 min 40 s for 35 cycles with a final extension at 72 °C for 10 min. Amplification products of about 1200 base pairs were isolated from agarose gel using Cleanup Standard kit (Evrogen) and sequenced on both strands by Evrogen [30]. The *CP* gene sequences assembled from the overlapping PCR fragments were deposited in GenBank under accession numbers MH346284−MH346316.

### 2.5. Whole Genome Sequencing

Genomic RNA from immunocaptured virus particles was employed for synthesis and amplification of cDNA libraries using Complete Whole Transcriptome Amplification Kit (WTA2, Sigma-Aldrich, St. Louis, MO, USA) as described previously [40]. The resulting libraries were subjected to the high-throughput sequencing on the Illumina MiSeq platform (isolates Ka7, Ka15, Ka23) or the 454 platform (Bg6, Bg26, Bg66, Pul). The contigs were assembled de novo using the SPAdes v.3.10.1 program [41]. The PPV-relevant contigs were identified by a BLASTn search (https://blast.ncbi.nlm.nih.gov/Blast.cgi) and aligned on the PPV genome that showed the greatest BLAST score. Appropriate primers were designed to fill the internal gaps. The 5’-terminal region was amplified using 5’RACE kit (Invitrogen, Carlsbad, CA, USA), following the manufacturer’s protocol. The corresponding PCR products were purified from agarose gel with Cleanup Standard kit (Evrogen) and sequenced in both directions using Evrogen facilities. The full-length genome sequences were deposited in GenBank under accession numbers MH311853–MH311859.

### 2.6. Sequence Analyses

Apart from sequences of the new isolates listed in Table 1, those belonging to the strains C, CR, CV, W, and EA were retrieved from GenBank and employed in the analyses. These were SwC (Y09851), SoC (AY184478), GC27 (KY221840), Volk143 (KJ787006), BY181 (HQ840518), BY101 (HQ840517), RU-17sc (KC020124), RU-18sc (KC020125), RU-30sc (KC020126), Fl-3 (MG736812), Kp8-1 (MG736813), Kp8-2U (MG736814), Pul-1 (MG736815), Pul-DS (MG736816), Tat-2 (MF447179), Tat-4 (MF447180), LV-141pl (HQ670746), LV-145bt (HQ670748), and EA-2005 (AM157175) as phylogenetic outgroup. Multiple alignments were carried out as described previously [5] using the ClustalW v.2.1 or an older version of this program included in the BioEdit v. 7.2.4 software [42]. Nucleotide (nt) and deduced amino acid (aa) sequence identities were determined employing the ClustalW algorithm implemented in the MegAlign v. 7.1.0. (DNASTAR Lasergene package, DNASTAR Inc., Madison, WI, USA). The PhyML 3.0 version of maximum likelihood algorithm [43] was used for phylogenetic analysis. Graphical tree presentation was performed by MEGA7 program [44] choosing “root on midpoint” option. The recombination detection program v.4.69 (RDP4) [45] was employed to search for recombination in the aligned complete genome sequences using default settings, with the exception that “sequences are linear” and “list events detected by >4 methods” options were chosen.

### 2.7. Western Blot Analysis

Western blot analysis of the PPV-C CP was carried out as described previously [30]. Leaf samples were homogenized in the 2× loading buffer using 1/15 ratio (*w*/*v*) and heated at 96 °C for 7 min. Proteins were resolved by 10% (*w*/*v*) SDS-PAGE and transferred to Immobilon-P membrane (Millipore, Burlington, MA, USA). Membrane was blocked for 1 h at room temperature with 5% (*w*/*v*) skim milk powder (Sigma-Aldrich, St. Louis, MO, USA), diluted in PBS, and probed with the antibody 5B at the 1/10,000 dilution. HRP-labeled anti-mouse IgG (W4021, Promega, Madison, WI, USA) was used as the secondary antibody (1/20,000 dilution). PBS with 0.1% (*v*/*v*) Tween 20 was used for membrane washing and dilution of immunospecific reagents. Detection was performed employing ECL kit (Promega).

### 2.8. Study on PPV-C Seed Transmission

Ripe fruits were harvested from cherries infected with the isolates Bg66 and Ka22. PPV was detected in skin of the collected fruits by RT-PCR. The flesh was removed and seeds were cold-stratified for germination in a moistened substrate for about six months. The virus in the seedlings was verified by RT-PCR using P1/P2 primers twice during a six months period after planting in pots.

## 3. Results

### 3.1. Detection and 3’-Terminal Sequence Analyses of the New PPV-C Isolates

Forty new PPV-C isolates were found in three geographically distant regions of European Russia (Table 1, Figure S1). All the isolates were readily detected by DAS-ELISA with the polyclonal antibodies to PPV (Table 2) and RT-PCR using the primers P1/P2. Based on the consistent results of RT-PCR with three pairs of the PPV-C-specific primers, they were identified as belonging to the strain C. No positive reaction in RT-PCR with primers to the strains D, M, CR, CV, or W was observed thus excluding mixed infections.

**Figure 1:**
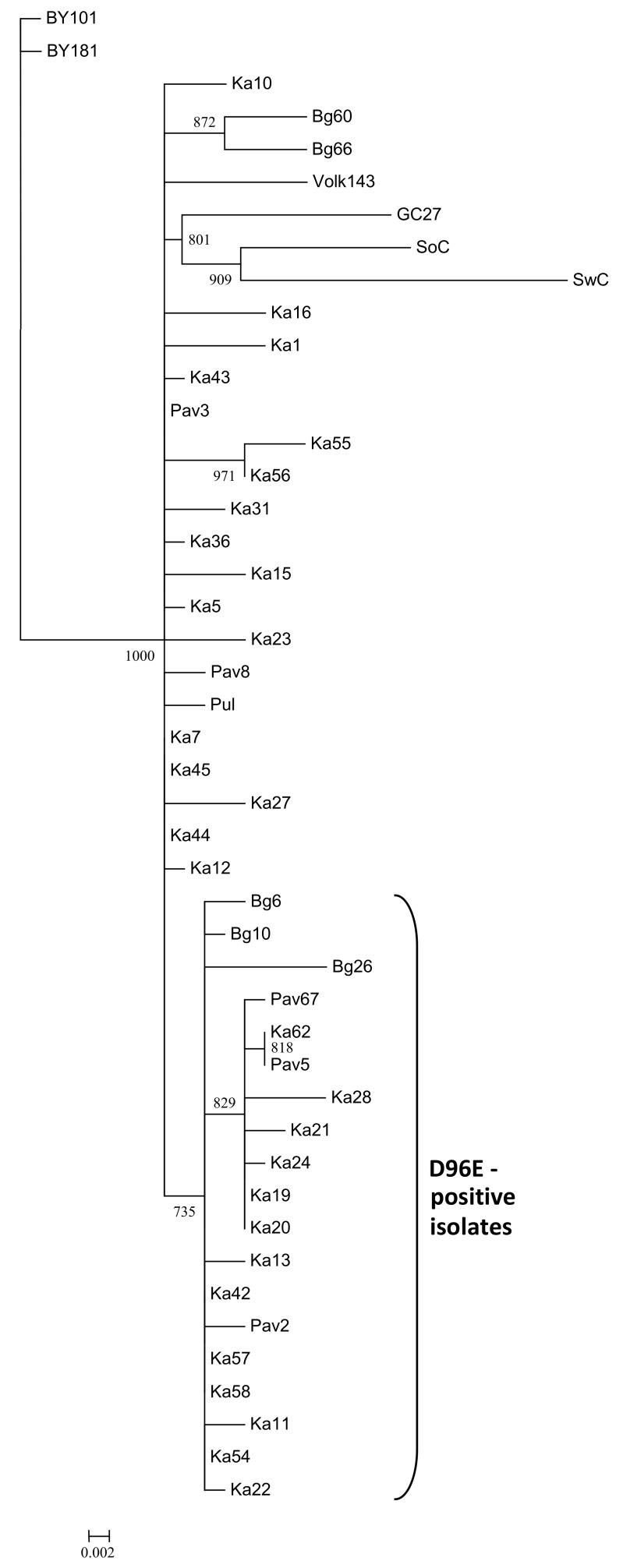
Phylogenetic tree based on the sequences of the coat protein gene of the *Plum pox virus* (PPV) strain C isolates. The tree was reconstructed using the PhyML 3.0 version of maximum likelihood algorithm [43]. Bootstrap values (>70%) are shown next to the corresponding nodes. The nodes with less than 70% bootstrap support were collapsed. GenBank accession numbers of the previously characterized isolates are indicated in the Materials and Methods section and those of new ones are presented in Table 1. The isolates bearing the D96E mutation are marked with a bracket. The scale bar indicates the number of substitutions per residue.

The strain C affiliation of the detected isolates was further confirmed by sequencing of the 3’-terminal genomic region followed by phylogenetic analysis of the *CP* gene. The new isolates were grouped together with the known PPV-C (Figure 1). The isolates Pav2, Ka16, and Bg26 found on cv. Tchereshnevaya in three distant localities were different from each other. Distinct isolates Ka42, Ka43, Ka44, and Ka45 were detected on the vegetative descendant of the seed-borne hybrid 102-8 suggesting their independent inoculation. On the contrary, the *CP* gene sequences of the Ka19 and Ka20 (from the vegetative offspring of the hybrid 33–64), as well as Pav5 and Ka62 (from cv. “Zarya Tatarii” maintained in the St. Petersburg and Tatarstan regions), were 100% identical to one another suggesting that corresponding mother plants seem to be the source of the virus. No geographical clustering of the PPV-C isolates was detected. Analysis of the deduced aa sequences of the CP of the new PPV-C isolates revealed 19 ones with the D96E mutation in the universal epitope (Table 2), indicating its broad distribution among PPV-C from three regions surveyed. According to phylogenetic analysis, the sequences of the *CP* gene of the D96E-positive isolates formed a separate cluster with 73.5% bootstrap support (Figure 1).

### 3.2. Serological Analysis

Isolates expressing the typical universal epitope DRDVDAG were recognized in TAS-ELISA with the antibody 5B whereas those bearing the D96E mutation were mostly negative (Table 2), which is consistent with the previous results of the investigation of the D96E-positive strain C and CR isolates [7,8,14]. At the same time, a number of samples demonstrated unexpected results in TAS-ELISA. The optical density in the K10 was obviously lower than in other isolates with the epitope DRDVDAG. The D100N substitution can potentially affect the antibody 5B binding due to differences in the side chains of asparagine (N) and aspartic acid (D). However, similar results were observed when analyzing the isolates Bg66, Ka5, Ka12, Ka27, Ka44, and Pav3 expressing the unchanged universal epitope. On the other hand, weak but distinguishable positive signals were detected for the isolates Ka42 and Ka62 bearing the D96E mutation.

**Figure 2:**
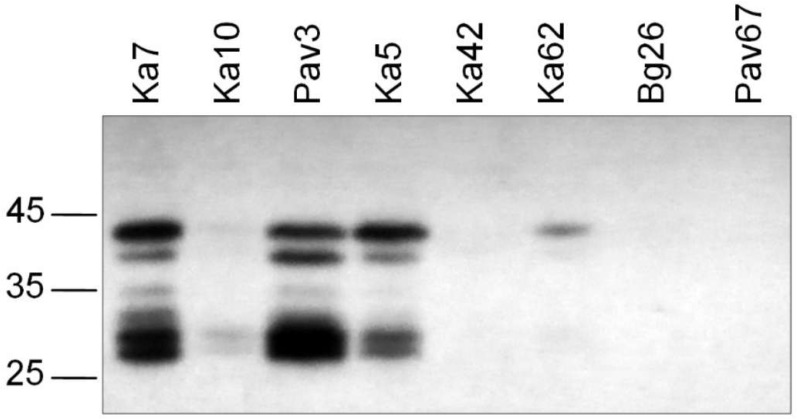
Western blot analysis of the selected PPV-C isolates differing in the sequence of the universal epitope. Arrows to the left of the picture indicate the positions of the molecular weight markers (kDa) (Thermo Scientific, Waltham, MA, USA).

**Figure 3:**
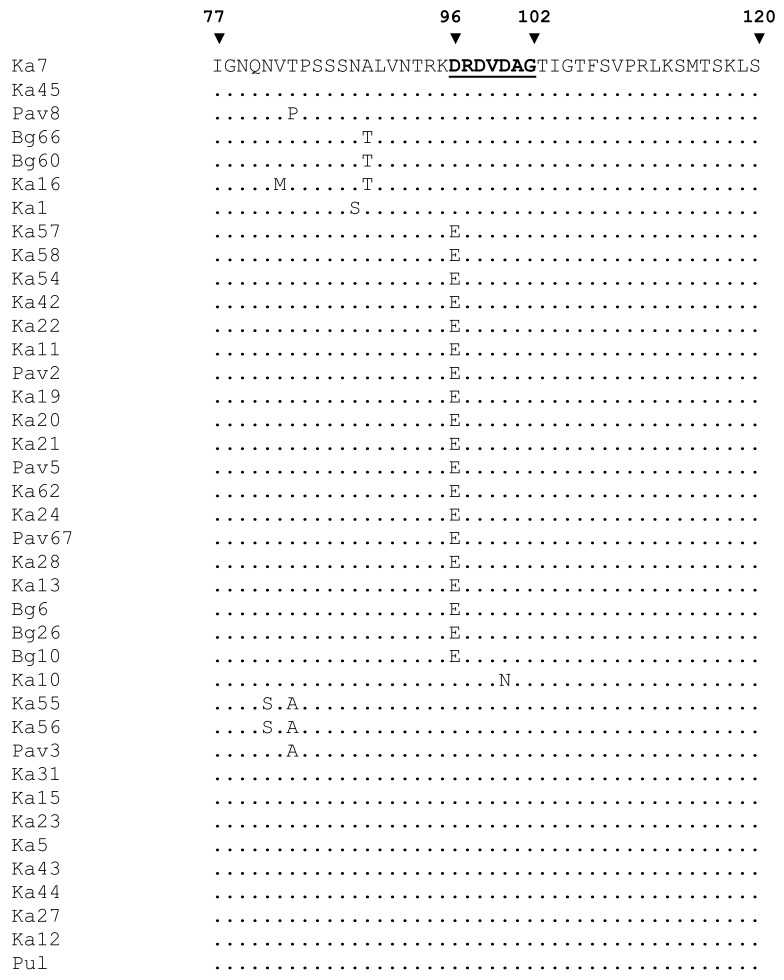
Multiple alignment of partial coat protein sequences of the Russian PPV-C. Numerals above the alignment indicate amino acid positions in the coat protein. The sequence of the universal epitope is highlighted.

Western blot analysis of selected samples, differing in the sequence of the universal epitope, demonstrated that the antibody 5B was capable of detecting a set of bands in the case of isolates Ka5, Ka7, and Pav3 containing the epitope DRDVDAG (Figure 2). This result shows that the denatured CP can be recognized with the antibody 5B as effectively as its counterpart in the intact virus particle thus indicating that conformation of the CP molecule is not crucial for the antibody 5B binding. Apparently, the uppermost band represents the full-length CP. Its molecular weight (MW) calculated in accordance with the electrophoretic mobility was about 41 kDa, which is well above the MW based on the amino acid composition (36.8 kDa). The atypical mobility may be due to glycosylation of the serine and threonine residues in the N-terminal domain with N-acetyl glucosamine as was previously shown for the strains D, M, and Rec by glycoprotein staining of Western blots [46] and mass spectrometry [47,48]. The lower bands are considered the products of cleavage of the surface-exposed N-terminal domain of the CP by cell proteases [49]. The antibody 5B can successfully bind to a partially digested CP as the universal epitope is located near the protease-resistant core and not affected by the proteolysis. In agreement with the TAS-ELISA results, the isolate Ka10 was weakly stained and Bg26 and Pav67 were not detected with the antibody 5B. As with TAS-ELISA, the D96E-positive isolates Ka42 and Ka62 were barely detected by Western blot analysis. The alignment of aa sequences around the universal epitope did not show any relevant substitutions in its immediate context capable of affecting the antibody 5B binding (Figure 3).

### 3.3. Analysis of Complete Genomes and Polyproteins

**Figure 4:**
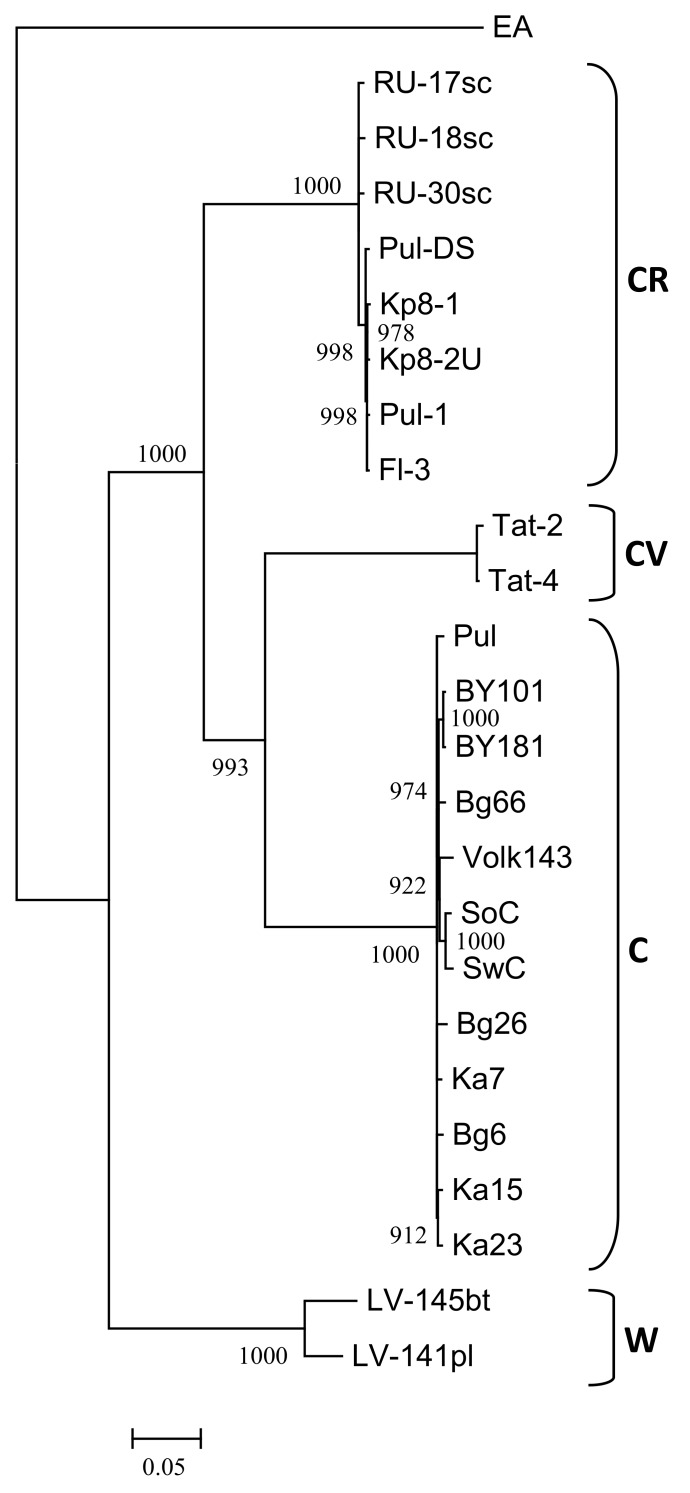

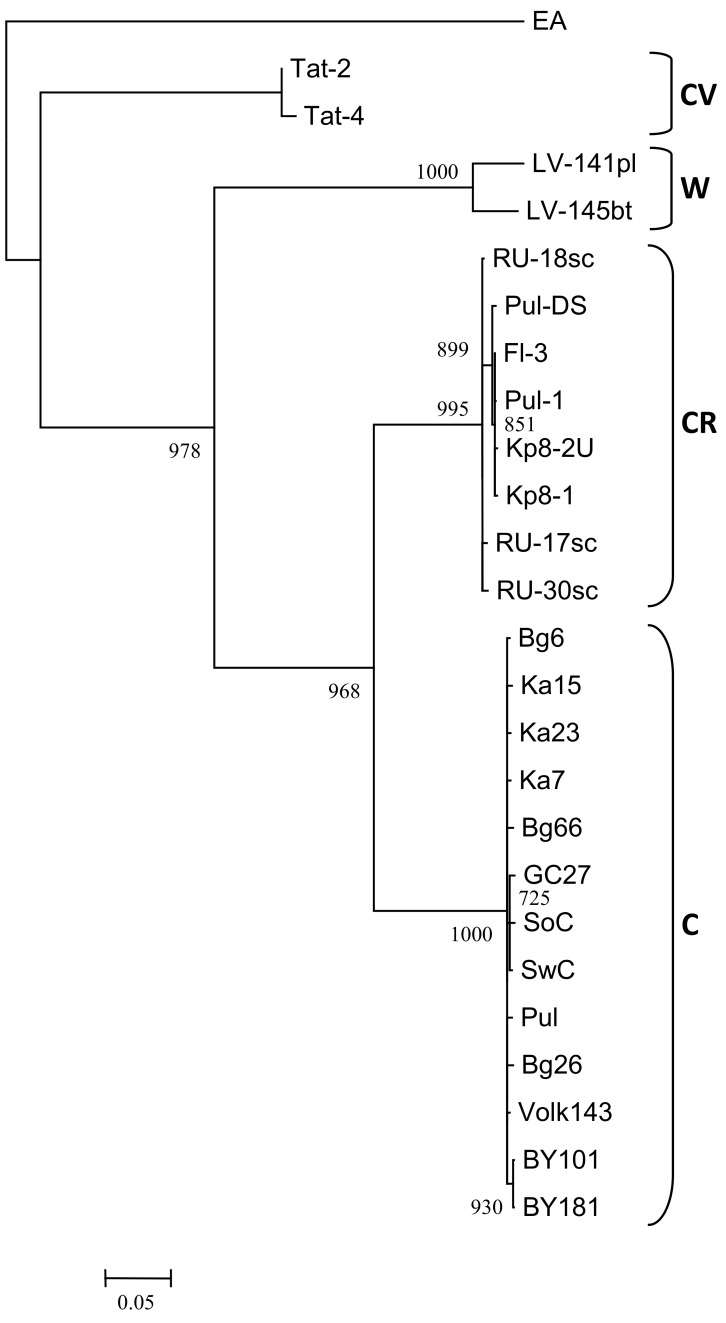
Phylogenetic analysis of full-length PPV strain C genome sequences (**A**) and putative recombinant sequences from nucleotide 664 to 1723 (**B**). The trees were reconstructed using the PhyML 3.0 version of maximum likelihood algorithm [43]. Bootstrap values (>70%) are shown next to the nodes. The nodes with less than 70% bootstrap support were collapsed. The strain affiliation showed on the right of the trees. GenBank accession numbers of the known isolates are indicated in the Material and Methods section and those of new ones are presented in Table 1. The scale bar indicates the number of substitutions per residue.

The nearly complete genome sequences of the isolates Ka7, Ka15, Ka23, Bg6, Bg26, Bg66, and Pul were assembled from one (Ka7, Ka23) to six (Pul) contigs (lengths from 202 to 9744 nt) covering 92.2 to 99.5% of the most closely related genome (BY181) with 7x (near the 5’-end of the genome) to 154x (near the 3’-end of the genome) minimal coverage. No heterogeneity was revealed in any next-generation sequencing (NGS)-analyzed samples, suggesting that each of them contains only a single PPV isolate. The remaining gaps were addressed by conventional Sanger sequencing using custom primers and 5’-RACE. There were no differences in sequences between the initial assembly and gap-covering PCR products in overlapping regions. The genomes of these isolates are composed of 9795 nt and encode polyproteins 3143 aa in length. Both nt and aa sequences are entirely collinear to the previously characterized PPV-C genomes. The ORF of PIPO [50] was identified at positions 2909 to 3226. The PPV-C isolates share 98.2 to 99.4% and 97.9 to 99.9% identities at the nt and aa levels, respectively. The phylogenetic analysis of new and known full-length PPV-C sequences together with several isolates belonging to the strains CR, CV, W, and EA was performed employing maximum likelihood algorithm. The genomes of the new PPV-C isolates are clustered with the known PPV-C with 100% bootstrap support (Figure 4A).

All putative cleavage sites and motifs associated with aphid transmission are identical in the polyproteins of the new isolates. Most of amino acid residues unique for the cherry-adapted strains [5,8] were found to be conserved, with a few exceptions. Alanine (A) at position 1022 (P3 protein) is substituted for valine (V) in the Ka23. Tyrosine (Y) at position 1415 (CI) is substituted for histidine (H) in the Bg6. Threonine (T) at position 2836 (CP) is substituted for isoleucine (I) in the Ka15. Serine at position 2919 (CP) is substituted for alanine in the newly sequenced isolate GC27 from Germany [22]. The mentioned positions probably should be excluded from the list of amino acids unique to cherry-adapted strains. In addition, based on generation of inferred ancestral sequences, Sanderson et al. [51] identified eight aa sites in the P1 (positions 117, 137, 158, 180) and VPg (positions 1949, 1957, 1970, 2038) proteins that seem to have undergone positive selection during PPV adaptation to cherries. All of them were conserved in the new PPV-C isolates. However, in the isolates Tat-2 and Tat-4 (strain CV), position 158 is occupied by glutamine (Q) instead of histidine (H) common for all other known cherry-adapted isolates [5], suggesting that this position is not important for virus adaptation to cherry. The isolate Ka15 was detected on naturally infected *P. tomentosa*. Five amino acid substitutions in P1 (Q36R), NIb (A2799V) and N-terminal domain of the CP (T2836I, D2860A, A2866T) distinguish it from sour cherry isolates. Whether these positions are related to the host range determination remains to be elucidated using more PPV-C isolates from downy cherry.

### 3.4. Recombination Analysis of the Complete Genome Sequences

**Figure 5:**
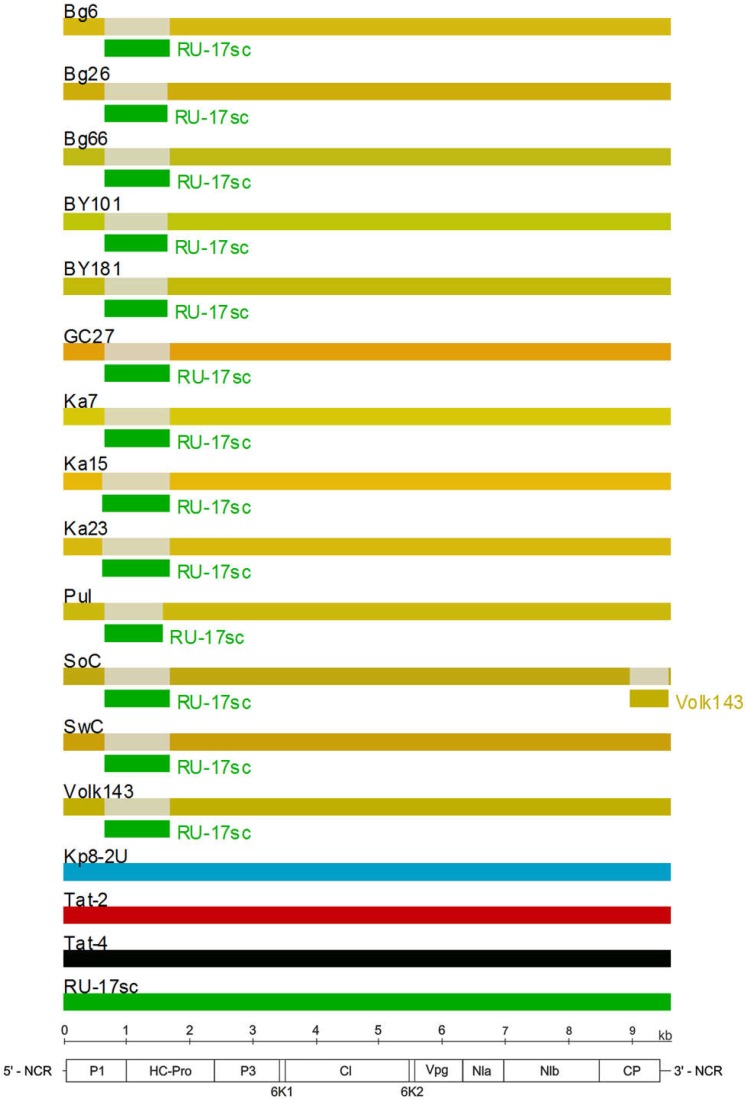
Analysis of recombination events in the alignment of thirteen full-length genomes of the PPV strain C isolates by the Recombinant Detection Program (RDP) v.4.69 [45]. Summarized results of the detection of the recombination events obtained by the six different methods implemented in the RDP4 are presented. The names of isolates are indicated above the long bars. The names of inferred minor parents are indicated next to short bars beneath the corresponding isolate bars. The map of the PPV genome and the ruler are presented in scale below the graph.

The RDP4 analysis detected the recombination event in the 5’-terminal region of all available PPV-C genomes (Figure 5). This event was confirmed by six different algorithms, implemented in the RDP4 suit, with statistically significant *P*-values (Table 3). The putative recombinant sequence encompasses the genomic segment from the (Cter)*P1* to the middle of the *HcPro* gene. Recombination breakpoints have been identified at nucleotide positions 625 to 664 and 1,653 to 1,723, depending on the isolate. The borders of the recombination event were verified by sequencing of the PCR products (≈450 nt long each) covering the putative breakpoint positions in the Bg6, Bg26, Bg66, Pul, Ka7, Ka15, and Ka23 genomes. Both sequences determined by the conventional Sanger method were identical to those obtained by the high-throughput sequencing, thus excluding the possibility that a chimeric sequences could be produced from two isolates co-infecting the host. The isolates Tat-4, belonging to the strain CV, and RU-17sc (PPV-CR) were inferred as major and minor parents, respectively. The results presented at Figure 5 was obtained employing the “minimal” alignment composed of thirteen full-length genomes of PPV-C as well as two ones of the strains CR (RU-17sc, Kp8-2U) and CV (Tat-2, Tat-4). The RDP4 analysis of the expanded alignment including, in addition, all other available full-length genomes of PPV-CR (RU-18sc, RU-30sc, Kp8-1, Pul-DS, Fl-3, Pul-1) and selected isolates of the strains W (LV-141pl, LV-145bt), EA (AM157175), D (AB545926, AY912056), and M (GR0019) generated similar results, although the breakpoint positions could be slightly shifted. On the contrary, exclusion of the Tat-2 and Tat-4 (inferred major parent) or RU-17sc (inferred minor parent), as well as any other PPV-CR, from the alignment makes this recombination event undetectable. This recombination event was confirmed by phylogenetic analysis. The inferred recombinant sequences of PPV-C clustered close to the corresponding genomic regions of the PPV-CR isolates, thus forming supercluster with 96.8% bootstrap support (Figure 4B). The trees based on the full-length genome sequences (Figure 4A) and the putative recombinant sequences (Figure 4B) were clearly incongruent, thus supporting recombination hypothesis. In addition, the putative recombinant sequence has, on average, 78.4 and 86.5% nt identities with the corresponding genomic region of the strains CV and CR, respectively, whereas the rest of the PPV-C genomes shares about 83.4% identity with both CV and CR strains. These data may also argue in favor of the recombination. Another intrastrain recombination event in the isolate SoC, displayed in Figure 5, has been detected and characterized previously [52].

### 3.5. The PPV-C Isolates Are Not Seed Transmitted

One hundred sixty-eight seedlings from PPV-C infected cherries were checked for the possibility of the virus transmission through seeds. Neither visible symptoms nor positive results in RT-PCR were observed up to six months after planting of the germinated seeds, suggesting that PPV-C is not transmitted through sour cherry seeds.

## 4. Discussion

In this work, forty new PPV-C isolates from three geographically distant regions of European Russia were characterized. The vast majority of them were discovered on sour cherry. Only one Russian PPV-C isolate was previously reported on sweet cherry [15]. Another strain C isolate was detected on naturally infected downy cherry (*P. tomentosa*). Downy cherry is the known woody indicator for PPV and can be experimentally infected with PPV-C [25,53]. However, to our knowledge, this is the first report of natural PPV-C infection of downy cherry under field conditions. Previous surveys of fruit orchards and private gardens as well as wild trees revealed the wide spread of PPV-C in European Russia [13,14,15,16]. In addition, the PPV-C isolate BarS-1 was detected on sour cherry in a private garden near the Barnaul town (Western Siberia) in the Asian part of Russia [17]. The results presented in this work may further contribute to clearer understanding of the prevalence and genetic diversity of PPV-C.

The high incidence of PPV-C in Russia is in obvious contrast with sporadic detection of this strain outside the country. This difference can be explained by assuming that PPV-C, or even all the cherry-adapted strains, originated in Russia, followed by its dissemination throughout European part of the country and occasional spreading into neighbor states. It should be noted that the isolate SoC first detected in Moldova is considered to be introduced from Russia together with infected sour cherry cultivars [9,25]. Remarkably, the Croatian PPV-C isolate was found to be identical with the SoC from Moldova [21].

The interstrain recombination detected in the 5’-terminal region among all the available full-length PPV-C genomes (Figure 5, Table 3) may shed more light on the evolution of this strain. The isolates belonging to the strains CV and CR were inferred as major and minor parents, respectively. Both strains were detected only in Russia and have a rather limited spread. The presence of this recombination event in all the studied PPV-C genomes suggest that it could occur in a precursor of the modern strain C isolates. Recombination could increase the fitness of its offspring and play an important role in the successful spread of PPV-C across the cherry plantings in Russia. The comparatively high *P*-values and high level of divergence of the presumed parents from each other indicate that this event should have happened a long time ago.

Seven new and six previously characterized PPV-C full-length genomes share 98.2 to 99.4% and 97.9 to 99.9% identities at the nt and aa levels, respectively, in agreement with previous data indicating low molecular heterogeneity of the strain C [15,22]. At the same time, the PPV-C population has been shown to be heterogeneous in terms that about half of the known isolates bear the D96E mutation while another one does not (Table 2). The D96E-positive isolates were revealed in three unrelated and geographically distant plantings. The vast majority of the PPV-C isolates bearing this mutation was not recognized by the antibody 5B in TAS-ELISA. The D96E mutation in the strain CR isolates was also found to affect their detection using this antibody [7,8]. Thus, this mutation may potentially compromise PPV detection in cherries due to false negative results when exploiting the antibody 5B. The reasons why this antibody recognizes the aspartic acid (D) residue but usually does not recognize the very similar glutamic acid (E) are unknown encouraging further investigations of the 5B/DRDVDAG interaction. For example, a lot of serine and threonine residues are concentrated around the universal epitope (Figure 3). Their glycosylation could interfere with the antibody 5B binding and explain the relatively low signals in TAS-ELISA for the isolates Bg66, Ka5, Ka12, Ka27, Ka44, and Pav3 (Table 2).

Interestingly, the sequences of the CP gene of the D96E-positive isolates were grouped together according to phylogenetic analysis (Figure 1). Similar results were previously obtained by studying *Potato virus* Y (PVY) [54]. A considerable part of the strain PVY^O^ isolates was shown to be recognized with the PVY^N^-specific monoclonal antibody 1F5 due to the single Q98R mutation in their CP. Phylogenetic analysis of complete genomes demonstrated that such isolates formed a separate subcluster O5 within the PVY^O^ strain with 100% bootstrap support. Except for serology, the PVY^O^-O5 group was not different from ordinary PVY^O^ [54]. Unlike PVY, full-length genomes of the Bg6 and Bg26 bearing the D96E mutation did not form a separate cluster (Figure 4A). It cannot be ruled out that in the future the segregation of two groups of PPV-C isolates can continue or, conversely, the D96E mutation can be eliminated from the population. It is worthy to note that the D96E mutation seems to be randomly distributed among the strain C isolates and does not appear to provide any selective advantage for their survival in sour cherry. For comparison, almost all the known PPV-CR isolates express the epitope ERDVDAG, while the PPV-CV CPs contain the DRDVDAG sequence.

No transmission of PPV-C through sour cherry seeds was detected. This result is in agreement with the previous data obtained with the apricot, peach, plum, and myrobalan seeds from PPV-D, -M, -Rec, and W-infected plants [55,56,57,58,59,60]. Meanwhile, the different isolates Ka5, Ka7, Ka10, Ka11, Ka21, Ka55, and Ka56 were found on seed-borne cherry trees (Table 1), suggesting that these plants could be independently infected through aphid transmission. The natural PPV-C vectors are unknown. The most efficient PPV vectors are considered *Myzus persicae*, *Aphis spiricola*, and *Hyalopterus pruni* [2]. The green peach aphid *M. persicae* and broadbean aphid *A. fabae* have been shown to transmit PPV-C isolates under laboratory conditions [11,18]. However, the colonizing *M. cerasi* was the only aphid species observed on cherry in the regions surveyed. Our attempts to transmit the isolates Pul, Ka13, Ka16, and Ka23 with *M. cerasi* from infected trees to sour cherry seedlings and *N. benthamiana* failed, in agreement with previous results of Labonne et al. [61].

## Figures and Tables

**Table 1:** New Russian *Plum pox virus* strain C isolates studied in this work.

Isolate	*Prunus cerasus* Cultivar/Hybrid	Type of Planting	Sampling Locality	Genomic Region Sequenced	GenBank Accession Number
Bg6	Lubskaya	Cultivar collection	Moscow	Complete genome	MH311853
Bg10	Saniya	Cultivar collection	Moscow	Coat protein	MH346284
Bg26	Tchereshnevaya	Cultivar collection	Moscow	Complete genome	MH311854
Bg60	Apukhtinskaya	Cultivar collection	Moscow	Coat protein	MH346285
Bg66	Bagryanaya	Cultivar collection	Moscow	Complete genome	MH311855
Ka1	Unknown	Abandoned cultivar collection 1	Tatarstan	Coat protein	MH346286
Ka5	Hybrid 12a-7-8	Cultivar test plot 1	Tatarstan	Coat protein	MH346287
Ka7	Truzhenitsa Tatarii × Rannyaya Sladkaya	Cultivar test plot 2	Tatarstan	Complete genome	MH311857
Ka10	Hybrid 34-86	Cultivar test plot 1	Tatarstan	Coat protein	MH346288
Ka11	Hybrid 98-19	Cultivar test plot 1	Tatarstan	Coat protein	MH346289
Ka12	Amorel Ten’kovskaya	Cultivar test plot 1	Tatarstan	Coat protein	MH346290
Ka13	Nizhnekamskaya	Cultivar test plot 1	Tatarstan	Coat protein	MH346291
Ka16	Tchereshnevaya	Cultivar test plot 1	Tatarstan	Coat protein	MH346292
Ka19	Hybrid 33-64	Cultivar test plot 1	Tatarstan	Coat protein	MH346293
Ka20	Hybrid 33-64	Cultivar test plot 1	Tatarstan	Coat protein	MH346294
Ka21	Hybrid 80-8	Cultivar test plot 1	Tatarstan	Coat protein	MH346295
Ka22	Tveritinovskaya	Cultivar test plot 1	Tatarstan	Coat protein	MH346296
Ka23	Sevast’yanovskaya	Cultivar test plot 1	Tatarstan	Complete genome	MH311859
Ka24	Pamyat Sakharova	Cultivar test plot 1	Tatarstan	Coat protein	MH346297
Ka27	Gilfanovskaya	Cultivar test plot 1	Tatarstan	Coat protein	MH346298
Ka28	Zonalnaya	Cultivar test plot 1	Tatarstan	Coat protein	MH346299
Ka31	Root offshoot	Cultivar test plot 2	Tatarstan	Coat protein	MH346300
Ka36	Morel Rannyaya	Private garden	Tatarstan	Coat protein	MH346301
Ka42	Hybrid 102-8/2	Cultivar test plot 1	Tatarstan	Coat protein	MH346302
Ka43	Hybrid 102-8/3	Cultivar test plot 1	Tatarstan	Coat protein	MH346303
Ka44	Hybrid 102-8/4	Cultivar test plot 1	Tatarstan	Coat protein	MH346304
Ka45	Hybrid 102-8/5	Cultivar test plot 1	Tatarstan	Coat protein	MH346305
Ka54	Root offshoot	Abandoned hybrid orchard	Tatarstan	Coat protein	MH346306
Ka55	Hybrid 1-4-16	Abandoned hybrid orchard	Tatarstan	Coat protein	MH346307
Ka56	Hybrid 1-5-2	Abandoned hybrid orchard	Tatarstan	Coat protein	MH346308
Ka57	Root offshoot	Abandoned hybrid orchard	Tatarstan	Coat protein	MH346309
Ka58	Root offshoot	Abandoned hybrid orchard	Tatarstan	Coat protein	MH346310
Ka62	Zarya Tatarii	Abandoned cultivar collection 2	Tatarstan	Coat protein	MH346311
Pav2	Tchereshnevaya	Cultivar collection	Pavlovsk	Coat protein	MH346312
Pav3	Shakirovskaya	Cultivar collection	Pavlovsk	Coat protein	MH346313
Pav5	Zarya Tatarii	Cultivar collection	Pavlovsk	Coat protein	MH346314
Pav67	Truzhenitsa Tatarii	Cultivar collection	Pavlovsk	Coat protein	MH346316
Pav8	Tukaevskaya	Cultivar collection	Pavlovsk	Coat protein	MH346315
Pul	Unknown	Wild tree	Moscow	Complete genome	MH311856
Ka15	***Prunus tomentosa***	Cultivar test plot 1	Tatarstan	Complete genome	MH311858

**Table 2:** Reactivity of *Plum pox virus* strain C isolates with polyclonal antibodies and monoclonal antibody 5B in double antibody sandwich (DAS) or triple antibody (TAS) enzyme-linked immunosorbent assay (ELISA), respectively.

Isolate	Optical Density at 405 nm ^1^		Universal Epitope ^4^
	DAS-ELISA ^2^	TAS-ELISA ^3^	
Bg6	2.42	0.20	**ERDVDAG**
Bg10	2.32	0.27	**ERDVDAG**
Bg26	2.80	0.21	**ERDVDAG**
Bg60	2.56	2.34	DRDVDAG
Bg66	2.69	1.09	DRDVDAG
Ka1	2.63	2.42	DRDVDAG
Ka5	2.63	1.36	DRDVDAG
Ka7	2.70	2.87	DRDVDAG
Ka10	2.57	0.78	DRDVNAG
Ka11	2.69	0.24	**ERDVDAG**
Ka12	2.76	1.45	DRDVDAG
Ka13	2.95	0.22	**ERDVDAG**
Ka15	2.23	2.35	DRDVDAG
Ka16	2.81	2.48	DRDVDAG
Ka19	2.85	0.24	**ERDVDAG**
Ka20	2.78	0.18	**ERDVDAG**
Ka21	2.68	0.24	**ERDVDAG**
Ka22	2.74	0.27	**ERDVDAG**
Ka23	3.12	2.87	DRDVDAG
Ka24	2.95	0.26	**ERDVDAG**
Ka27	2.78	1.74	DRDVDAG
Ka28	3.07	0.24	**ERDVDAG**
Ka31	2.82	2.66	DRDVDAG
Ka42	2.54	0.66	**ERDVDAG**
Ka43	2.71	2.20	DRDVDAG
Ka44	1.95	1.59	DRDVDAG
Ka45	2.66	2.55	DRDVDAG
Ka54	2.40	0.24	**ERDVDAG**
Ka55	2.54	2.18	DRDVDAG
Ka56	2.76	2.34	DRDVDAG
Ka57	2.57	0.23	**ERDVDAG**
Ka58	2.68	0.29	**ERDVDAG**
Ka62	2.65	0.69	**ERDVDAG**
Pav2	2.78	0.22	**ERDVDAG**
Pav3	2.65	1.46	DRDVDAG
Pav5	2.72	0.21	**ERDVDAG**
Pav67	2.63	0.23	**ERDVDAG**
Pav8	2.89	2.56	DRDVDAG
Pul	2.74	2.96	DRDVDAG
PPV-free cherry ^5^	0.21	0.24	

^1^ From triplicate; ^2^ Reagent set SRA 31505 (Agdia, USA); ^3^ K-10B kit (Agritest, Italy); ^4^ Amino acids 96 to 102 of the coat protein. The epitope ERDVDAG is in bold; 5. Negative control.

**Table 3:** Recombination events inferred in *Plum pox virus* strain C complete genomes by the Recombination Detection Program v.4.69 (RDP4).

Event Number	Recombinant	Beginning Breakpoint	Ending Breakpoint	Major Parent	Minor Parent	*p* Values Calculated with Seven Methods Implemented in RDP4						
						RDP	Geneconv	Bootscan	Maxchi	Chimaera	SiScan	3Seq
1	Bg6	664	1723	Tat-4	RU-17sc	3.6 × 10^−7^	Not detected	7.5 × 10^−5^	2.7 × 10^−4^	8.1 × 10^−4^	1.3 × 10^−22^	2.1 × 10^−8^
	Bg26	664	1703									
	Bg66	664	1723									
	BY101	664	1703									
	BY181	664	1703									
	GC27	649	1723									
	Ka7	664	1723									
	Ka15	625	1723									
	Ka23	625	1723									
	Pul	649	1653									
	SoC	664	1723									
	SwC	664	1723									
	Volk143	664	1723									
2	SoC	9096	9756	SwC	Volk143	2.0 × 10^−3^	7.9 × 10^−3^	8.1 × 10^−3^	1.2 × 10^−3^	9.1 × 10^−4^	7.4 × 10^−3^	2.4 × 10^−3^

## Supplementary Materials

The following are available online at http://www.mdpi.com/1999-4915/10/9/450/s1, Figure S1: Geographical localities (blue arrows) of *Plum pox virus* isolates studied in this work.
