# Supplementary figures and images for: Sequence Analysis of Plum pox virus Strain C Isolates from Russia Revealed Prevalence of the D96E Mutation in the Universal Epitope and Interstrain Recombination Events
Source: Viruses. 2018 Aug 23;10(9):450. doi: 10.3390/v10090450 (PMC6164383; doi:10.3390/v10090450)

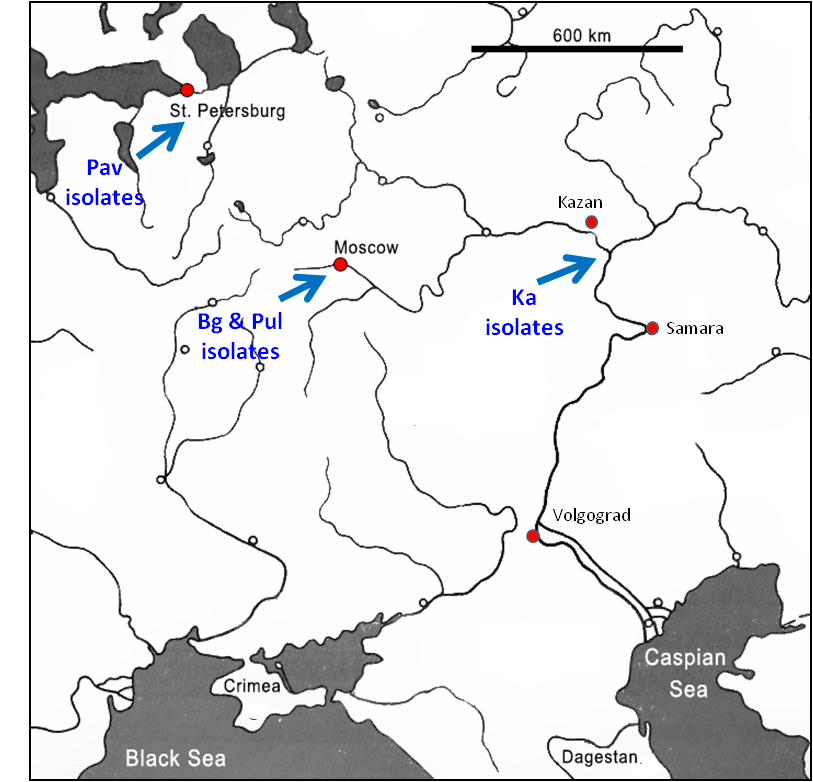

Supplement: Supplementary file 1 [file viruses-10-00450-s001.zip › viruses-334188 final supplementary.png]
